# Low PAPP-A Levels and Growth in Twin Pregnancies

**DOI:** 10.3390/life16010149

**Published:** 2026-01-16

**Authors:** Ioakeim Sapantzoglou, Dimitrios Papageorgiou, Afroditi Maria Kontopoulou, Christina Karasmani, Angeliki Rouvali, Afroditi Pegkou, Maria Simou, Ioannis Pafilis, Athina Souka, Marianna Theodora, Panagiotis Antsaklis, Georgios Daskalakis

**Affiliations:** 1 1st Department of Obstetrics and Gynecology, National and Kapodistrian University of Athens, 11527 Athens, Greece; 2 Department of Gynecology, Athens Naval and Veterans Hospital, 11521 Athens, Greece; 3 Iatriki Embryou-Ioannis Pafilis, 10676 Athens, Greece

**Keywords:** PAPP-A, twin pregnancy, multiple gestations, first-trimester analytes, SGA, FGR, birthweight

## Abstract

Background/Objectives: It is well established in the modern literature that newborns delivered from multiple gestations are more predisposed to low birthweight in comparison to their singleton equivalents. In this study, we sought to explore the potential of first-trimester biochemical (PAPP-A and free β-hCG) and biophysical indices (uterine artery Doppler) to predict low birthweight in one or both twins. Methods: This is a retrospective cohort analysis of 400 twin viable pregnancies presenting for routine first-trimester assessment in four fetal medicine centers between 2014 and 2025. The examination included the recording of maternal demographic characteristics and medical history, the assessment of markers of aneuploidy and the fetal anatomy, the measurement of mean arterial pressure, the assessment of uterine arteries and the measurement of serum concentration of PAPP-A and free β-hCG. The evaluated outcomes included BW ≤ 3rd centile and BW ≤ 10th centile in one or both twins based on local population birthweight reference charts. Results: The study cohort consisted of 400 twin pregnancies. BW ≤ 3rd centile in one or both twins was reported in 1.5 and 3.8% of cases, respectively, and there was no association of BW ≤ 3rd centile with any of the studied parameters. BW ≤ 10th centile in one or both twins was reported in 14.8 and 9.8% of the cases, respectively. PAPP-A MoM values were significantly lower in cases complicated by BW ≤ 10th centile in one and in both twins, remaining statistically significant even after the appropriate multiple logistic regression. PAPP-A MoM demonstrated statistically significant but low prognostic value for BW ≤ 10th centile in either one or both twins. Conclusions: Low PAPP-A levels were associated with BW ≤ 10th centile in one and both twins and its significant value as a risk marker was demonstrated. Higher PAPP-A MoM halves the risk of having at least one twin with low BW. Other maternal biophysical and biochemical indices did not seem to be predictive of low birthweight.

## 1. Introduction

Multiple gestations exhibit a higher susceptibility to fetal growth restriction (FGR) and low birthweight (BW) [1], with the incidence of FGR and small for gestational age (SGA) in twins being strongly correlated with the method of evaluation applied. The literature presents various conflicting definitions of FGR in twin pregnancies, which further impedes the synthesis of their findings [2,3]. Furthermore, utilizing singleton growth charts in twin pregnancies seems to exaggerate the aberrant growth rate, and current clinical evidence from multiple research groups indicates that these charts might be excessively confusing [4]. Nevertheless, irrespective of the FGR definition or the chart used, prior research has demonstrated that twin pregnancies are linked to increased perinatal morbidity and mortality relative to singleton pregnancies [5]. It is also well established that newborns delivered from multiple gestations are more predisposed to low birthweight in comparison to their singleton equivalents [6,7].

First-trimester assessment in twin pregnancies aims at assessing the risk of chromosomal and structural abnormalities. In contrast to the singleton pregnancies, screening for feto-placental diseases (preeclampsia/growth restriction) has not proven to be equally helpful in twins [8]. It is well established that uncomplicated twin pregnancies, compared with singletons, demonstrate lower uterine artery (Ut-A) PI and higher mean arterial pressure (MAP), placental growth factor (PlGF) and pregnancy-associated plasma protein A (PAPP-A) levels [9]. However, PAPP-A levels in twin pregnancies demonstrated nonsignificant alterations between uncomplicated pregnancies and those that eventually developed preeclampsia (PE). As such, its addition into the first-trimester screening for preeclampsia does not seem to provide any additional predictive benefit when maternal factors, Ut-A PI, MAP and PlGF are already in use [8,9].

In view of the fact that fetal smallness or FGR in twin pregnancies often occurs along with hypertensive disorders of pregnancy leading, for example, to iatrogenic preterm birth, the literature has suggested a potential association with inadequate placental perfusion [10]. While several studies have reported lower levels of first-trimester PAPP-A in twin pregnancies complicated by growth abnormalities in one or both fetuses, the actual predictive ability of this marker is still unclear [11,12]. As such, the clinical impact of the first-trimester assessment in the subsequent development of growth abnormalities in twin pregnancies remains uncertain.

Given the inconsistent findings of previous studies and the unavailability of PlGF in everyday practice within several fetal medicine settings, our study sought to explore the potential of first-trimester biochemical (PAPP-A and free β-hCG) and biophysical indices (uterine artery Doppler) to predict low birthweight in one or both twins.

## 2. Materials and Methods

This is a retrospective cohort analysis of twin viable pregnancies presenting for routine first-trimester examination at 11–14 weeks in four fetal medicine centers between 2014 and 2025. Exclusion criteria included demise of one twin, monoamniotic twins, chromosomal and major fetal structural abnormalities, twin-to-twin transfusion syndrome (TTTS), twin anemia-polycythemia sequence (TAPS), cases that underwent cord ablation or selective termination and loss to follow-up.

The first-trimester visit included the recording of maternal demographic characteristics and medical history; the ultrasound examination for assessment of the nuchal translucency, secondary markers of aneuploidy and the fetal anatomy; the measurement of mean arterial pressure; the assessment of uterine arteries by transabdominal ultrasound; and the measurement of serum concentration of PAPP-A and free β-hCG by one of two automated and validated biochemical analyzers: Kryptor (Thermo Fisher Scientific, Clinical Diagnostics, Brahms GmbH, Henningsdorf, Germany) or Cobas (Roche Diagnostics, Basel, Switzerland). Subsequently, the concentrations of the first-trimester biochemical analytes were converted to multiples of the median (MoM) utilizing Astraia softwareVersion 29.1.2 (Astraia Gmbh, Germany), adjusted for multiple pregnancy, to exclude the influence of factors such as gestational age, weight, race, conception method, medical problems and obstetric history components related to the individual being assessed.

Gestational age was determined based on the last menstrual cycle, which was validated or adjusted by measuring the fetal crown–rump length of the larger twin during the first-trimester ultrasound. In cases of assisted reproductive technology (ART), the dating was based on the date of embryo transfer or according to the estimated due date (EDD) provided by the ART center. Chorionicity was determined using ultrasonographic criteria: lambda or T-sign for dichorionic (DCDA) and monochorionic (MCDA) twins, respectively [13].

The national management policy of twin pregnancies is in accordance with the ISUOG guidelines [13,14]. Uncomplicated DCDA pregnancies are offered a first-trimester scan, a second-trimester anomaly scan and scans every 4 weeks thereafter which include the assessment of fetal growth, amniotic fluid and fetal Dopplers (umbilical artery PI—UA PI, middle cerebral artery PI—MCA PI, middle cerebral artery Peak Systolic Velocity—MCA PSV, ductus venosus PI—DV PI). Uncomplicated MCDA pregnancies are offered a first-trimester scan and scans every 2 weeks after 16 weeks of pregnancy. The gestational age for delivery of uncomplicated DCDA twins is considered to be between 37 + 0 and 37 + 6 weeks, and for uncomplicated MCDA twins between 36 + 0 and 36 + 6 weeks. In DCDA pregnancies, the follow-up in cases of SGA or FGR in one (selective SGA/FGR—s-SGA/s-FGR) or both twins is similar to that of singleton pregnancies [15]. In MCDA pregnancies complicated by SGA/FGR of both twins or s-SGA/s-FGR, there is no clear management guideline but follow-up, management options (conservative management followed by early delivery, laser ablation or selective termination of the growth-restricted twin in order to protect the cotwin) and delivery timing are mostly based on the fetal Doppler assessment [13,14]. However, as already stated above, cases that underwent cord ablation or selective termination were excluded from the study for our sample to be as homogenous as possible.

The pregnancy outcome was ascertained after communication with the physician or the mother. The evaluated outcomes included BW ≤ 3rd centile and BW ≤ 10th centile in one or both twins based on local population birthweight reference charts (unpublished data—Supplementary Materials).

### Statistical Analysis

Quantitative variables were expressed as mean values (standard deviation) and as median (interquartile range), while categorical variables were expressed as absolute and relative frequencies. For the comparison of proportions, chi-square and Fisher’s exact tests were used. Quantitative variables were tested for normality using the Kolmogorov–Smirnov criterion. If the normality assumption was satisfied for the comparison of means between two groups, Students’ *t*-tests were used. The Mann–Whitney test was used for the comparison of continuous variables between two groups when the distribution was not normal. Logistic regression analysis in a stepwise method (*p* for entry 0.05, *p* for removal 0.10) was used to identify independent factors associated with low BW. Adjusted odds ratios (OR) with 95% confidence intervals (95% CI) were computed from the results of the logistic regression analyses. All reported *p*-values are two-tailed. The prognostic value of PAPP-A MoM was evaluated via ROC analysis. Sensitivity, specificity, negative prognostic values (NPVs) and positive prognostic values (PPVs) with their 95% confidence intervals (95% CI) were calculated for optimal cut-offs. The area under the curve (AUC) was also calculated. Statistical significance was set at *p* < 0.05 and analyses were conducted using SPSS statistical software (version 27.0).

## 3. Results

The study cohort consisted of 400 twin pregnancies. The demographic characteristics are presented in Table 1. Spontaneous conception occurred in 41.8% (167/400) of the included sample and 87.3% (349/400) of the included twin pregnancies were dichorionic. Baseline characteristics were compared in patients with missing vs. non-missing data and there were no significant differences (*p* > 0.05).

BW ≤ 3rd centile in one or both twins was reported in 1.5 and 3.8% of cases, respectively. There was no association of BW ≤ 3rd centile with any of the studied parameters (Table 2).

BW ≤ 10th centile in one or both twins was reported in 14.8 and 9.8% of cases, respectively (Table 3). PAPP-A MoM values were significantly lower in cases complicated by BW ≤ 10th centile in one and in both twins. Free β-hCG MoM was also found to be significantly lower in cases with BW ≤ 10th centile in one twin.

In multiple logistic regression analysis, only PAPP-A MoM was significantly associated with BW ≤ 10th centile in one twin (OR = 0.92; *p* = 0.004) or in both twins (OR = 0.94; *p* = 0.041) (Table 4).

**Figure 1:**
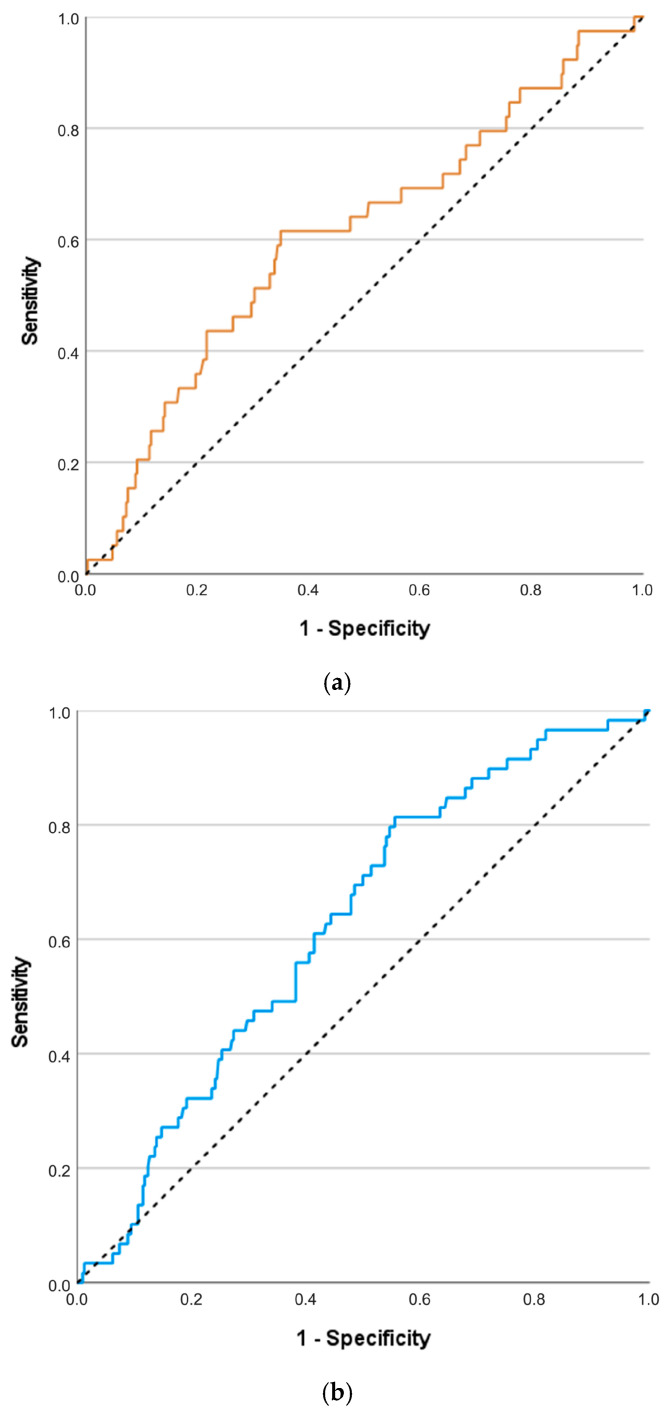
ROC curves for the prognostic value of PAPPA-MoM regarding BW ≤ 10th centile in one (**a**) and in both twins (**b**). Black dashed line: reference line.

PAPP-A MoM had statistically significant but low prognostic value for BW ≤ 10th centile in any or both twins (Table 5). The ROC curves for the prognostic value of PAPP-A MoM regarding BW ≤ 10th centile are presented in Figure 1.

## 4. Discussion

### 4.1. Principal Findings of Our Study

Low PAPP-A levels were associated with BW ≤ 10th centile in one and both twins, with PAPP-A’s predictive ability being clear but limited. Higher PAPP-A MoM halves the risk of having at least one twin with low BW and the optimal cut-off was estimated at 1.408 MoM. PAPP-A demonstrated an existent but limited predictive value. Maternal biophysical indices (MAP, uterine Doppler studies) were not predictive of low birthweight.

### 4.2. Comparison to Existing Literature

In singleton pregnancies, the association of low PAPP-A levels with adverse pregnancy outcomes and growth restriction or fetal smallness is well established [16,17]. The association appears to be so strong that several guidelines advocate a tight and strict monitoring of pregnancies complicated by low PAPP-A levels (below the 5th centile or below 0.415 MoM), with additional ultrasound assessments and regular fetal surveillance being recommended [18,19]. The correlation of PAPP-A with fetal growth in singleton pregnancies has also been emphasized by its inclusion, along with maternal factors, prior obstetric history and other first-trimester biophysical (MAP, Ut-A Doppler) and biochemical (placental growth factor—PlGF) analytes, in risk assessment models for the prediction of subsequent SGA and FGR [20,21,22]. However, such a risk assessment model for twin pregnancies is yet to be formulated and the association of PAPP-A levels with fetal smallness or growth restriction is still unclear as studies have produced contradictory results [23]. It needs to be underlined, though, that first-trimester biomarkers, even in singleton pregnancies, have not demonstrated a significant predictive capacity for other perinatal complications such as preterm delivery (PTD) or preterm premature rupture of membranes (PPROM), a fact that may be explained by the inherent limitations of those markers, limitations that may be exacerbated in twin pregnancies [23,24].

It needs to be highlighted that the modern literature includes only a small number of studies that have investigated the potential association of first-trimester PAPP-A with birthweight in twin pregnancies. The study by Saletra-Bielinska et al., which investigated the potential association of PAPP-A < 10th centile with the subsequent development of perinatal complications, did not manage to demonstrate an association between low PAPP-A levels and either BW < 10th centile or severe BW discordance among the newborns [25]. Contrary to the above, the study by Kim et al., which enrolled a total of 524 twin pregnancies, demonstrated that newborns with BW < 10th centile had lower PAPP-A levels compared to appropriately grown newborns. In their study, PAPP-A levels remained independently associated with low birthweight after the appropriate adjustments [26]. Furthermore, a recent study of 466 twin pregnancies revealed a significant association of PAPP-A levels < 10th centile with FGR < 3rd centile in one or both fetuses [11]. The same study group identified low PAPP-A levels and increased Ut-A PI as independent risk factors for FGR < 3rd centile and FGR < 3rd centile combined with preterm birth < 32 weeks of gestation [12]. However, even though several of the above-mentioned studies, along with our results, have identified PAPP-A as a risk marker for fetal/newborn smallness, it should be noted that its predictive value remains limited, especially in view of the emerging role of PlGF. Several well-conducted large datasets have demonstrated PlGF’s superior role—compared to PAPP-A—in predicting the subsequent development of PE [8,9,27]. To be more precise, the recently published preliminary results from the IPSISS (Implementing Preeclampsia Screening in Switzerland) study demonstrated statistically significant lower PlGF levels in pregnancies complicated with PE, while PAPP-A was not proven to be significantly lower [27]. However, they were unable to calculate the detection rates (DRs) and screen positive rates (SPRs) between different combinations of first-trimester markers due to the use of low-dose aspirin in high-risk women. On the other hand, Francisco et al., in their cohort of 35,948 singleton and 1100 twin pregnancies, demonstrated increased measurements of MAP and Ut-A PI and decreased serum PLGF levels at 11–13 weeks’ gestation in pregnancies that were subsequently complicated by PE. According to the results of this study, the combination of maternal factors, MAP, Ut-A PI and PlGF provided the best screening performance for PE. In this combination, adding PAPP-A offered no additive value in the screening performance [9]. Similar results were demonstrated by the study of Benko et al. [8], in which the authors combined the dataset of Fracisco et al. with the dataset of EVENTS (Early vaginal progesterone for the preVention of spontaneous preterm birth iN TwinS) trial [28], showing the superiority of PlGF—compared to PAPP-A—when it was combined with maternal factors, MAP and Ut-A PI. Such a finding is aligned with the results of singleton cohorts [29].

In view of the value of Ut-A PI as a risk factor for low BW, our results differ to some of the already published studies, further enforcing the already conflicting literature on the issue. Rizzo et al. showed elevated Ut-A PI in twin pregnancies that developed early PE and SGA of both twins without significant changes in terms of late PE or s-SGA [30]. However, larger cohorts do not confirm these findings and are aligned with our results, demonstrating the limited predictive value of first-trimester Ut-A PI, a fact that may be attributed to a different underlying mechanism causing PE and growth abnormalities in twins other than placental underperfusion [31,32].

### 4.3. Clinical Significance

The findings of our study, similar to the results of prior research, seem to further confirm the crucial regulatory role of PAPP-A in placental development, volume and function and therefore its importance on fetal growth [33,34]. The identification of low first-trimester PAPP-A levels may act as an early risk assessment tool to guide and enhance the management and follow-up of twin pregnancies, proving to be as useful as in singleton pregnancies [11,23,35]. Therefore, low PAPP-A levels seem to be useful in the risk stratification of twin pregnancies as well, potentially leading to the improvement of subsequent surveillance and care. However, it should be underlined that, based on our findings, PAPP-A demonstrated a relatively limited predictive ability, and for solid conclusions to be extracted and for accurate and clinically useful predictive models to be formulated, more studies with richer datasets are required.

### 4.4. Strengths and Limitations

The principal strength of the study is that it includes a large non-selected cohort that presented for routine first-trimester assessment and is therefore representative of the twin obstetric population. All examiners had fetal medicine training and the pregnancies were followed-up with serial ultrasound examinations. Our research demonstrated the value of PAPP-A in predicting fetal smallness, acting as a red flag for more enhanced antenatal surveillance.

However, there are several limitations that need to be acknowledged. Our results did not include the measurement of the PIGF, which has been shown to have superior predictive ability for growth restriction compared to PAPP-A in singleton pregnancies [27,36]. The cohort included both DCDA and MCDA pregnancies, which differ in the prevalence of fetal growth complications, but the number of monochorionic twins was not large enough to perform a sub-analysis based on the chorionicity. Furthermore, the small sample size and low event count represent limitations, as they may lead to statistical instability and wider confidence intervals. Consequently, our findings should be considered exploratory. Additionally, MAP measurements were available in 243 cases within our cohort, a fact that might have influenced our final results, underpowering our analysis. Since there is no national policy for the assessment of the risk for PE or SGA/FGR in the first trimester in twin pregnancies (given the limited literature data), the measurement of MAP is not part of our standard practice in twin pregnancies and the measurements that were eventually documented and included in our study were actually taken as part of another maternal-related research study. Furthermore, almost all women were using aspirin. In our cohort, 83.5% (334/400) of women were on aspirin. All of the included IVF twin pregnancies (234/400) and 60.2% (100/166) of the spontaneously conceived ones were given aspirin. The typical dose provided was 160 mg once per day. It was usually prescribed as soon as the presence of the twin pregnancy was confirmed (typically at around 7–8 weeks of gestational age, the time period in which the first antenatal ultrasound assessment is performed in pregnant women in our country), several weeks before attending their first-trimester screening assessment at the included fetal medicine centers. Aspirin administration is known to have an impact on the subsequent reduction in Ut-A PI and, thus, a positive effect on placental perfusion [37]. As such, such an improvement in placental perfusion may lead not only to lowering the incidence of subsequent PE, but it may also alter the rates of growth impairment, potentially affecting the screening performance of uterine artery Doppler in our study.

## 5. Conclusions

Low PAPP-A levels were significantly associated with low birthweight in one and in both fetuses, and its value as a risk marker was demonstrated. Contrary to singleton pregnancies, multiple pregnancies lack accurate risk assessment models for common pregnancy complications. However, given the fact that multiple pregnancies are susceptible to increased rates of adverse pregnancy outcomes, future studies need to focus on investigating and formulating predictive models to guide management and clinical decisions.

## Figures and Tables

**Table 1:** Maternal demographic characteristics and biochemical and biophysical parameters of the study sample (*n* = 400 twin pregnancies).

			*n*	%
Maternal characteristics	Conception	Spontaneous	167	41.8
		IVF	233	58.3
	Chorionicity	Dichorionic	349	87.3
		Monochorionic	51	12.8
	BMI levels	Underweight	10	2.5
		Normal	220	55.0
		Overweight	111	27.8
		Obese	59	14.8
	Nulliparous		289	72.3
	Smoking		37	9.3
	Pre-existing diabetes		11	2.8
	Pre-existing hypertension		3	0.8
	Systemic lupus erythematosus		3	0.8
	Antiphospholipid syndrome		2	0.5
	History of preeclampsia		2	0.5
	History of low birthweight		0	0.0
			Mean (SD)	Median (IQR)
	Age		35.6 (4.3)	35.2 (33–38.4)
	Weight		69.4 (14.6)	66.5 (59–76)
	Maternal height		166.0 (5.9)	165 (162–170)
	BMI (1st trimester)		25.2 (5.0)	23.9 (21.6–27.7)
	Birthweight (gr)		2275.7 (539.7)	2325 (2000–2607.5)
Biochemical parameters	PAPP-A MoM		1.34 (0.65)	1.23 (0.89–1.67)
	Free β-hCG MoM		1.20 (0.78)	1.02 (0.73–1.5)
Biophysical parameters	MAP ^1^		89.0 (8.2)	88.8 (83.3–93.8)
	Mean Uterine Artery PI ^2^		1.48 (0.44)	1.42 (1.18–1.72)

^1^ available in 243 participants, ^2^ available in 374 participants. Abbreviations: BW: birthweight, BMI: body mass index, underweight: BMI < 18.5, overweight: BMI 25–30, obese: BMI > 30, MAP: mean arterial pressure.

**Table 2:** Association of low BW ≤ 3rd centile with maternal characteristics and biochemical and biophysical parameters.

		BW ≤ 3rd in One Twin		*p*-Value	BW ≤ 3rd Both Twins		*p*-Value
		No (*n* = 385; 96.3%)	Yes (*n* = 15; 3.8%)		No (*n* = 394; 98.5%)	Yes (*n* = 6; 1.5%)	
		*n* (%)	*n* (%)		*n* (%)	*n* (%)	
Age, mean (SD)		35.5 (4.2)	37.3 (5.6)	0.111 ‡	35.6 (4.3)	34.7 (4.4)	0.627 ‡
Weight, mean (SD)		69.5 (14.6)	66.5 (14)	0.435 ‡	69.3 (14.3)	76.7 (28.5)	0.219 ‡
Height, mean (SD)		166.1 (6)	164.9 (4.1)	0.470 ‡	166 (5.9)	167 (6.5)	0.684 ‡
BMI, mean (SD)		25.2 (5)	24.4 (4.8)	0.547 ‡	25.1 (5)	27 (8.1)	0.358 ‡
Birthweight, mean (SD)		2287.0 (540.3)	1986.8 (448.9)	0.034 ‡	2280.7 (541.5)	1945.0 (253.7)	0.022 ‡
BMI	Underweight/Normal	222 (96.5)	8 (3.5)	0.815 ++	226 (98.3)	4 (1.7)	0.183 ++
	Overweight	107 (96.4)	4 (3.6)		111 (100)	0 (0)	
	Obese	56 (94.9)	3 (5.1)		57 (96.6)	2 (3.4)	
Chorionicity	Dichorionic	335 (96)	14 (4)	0.704 ++	343 (98.3)	6 (1.7)	>0.999 ++
	Monochorionic	50 (98)	1 (2)		51 (100)	0 (0)	
Conception	IVF	223 (95.7)	10 (4.3)	0.500 +	230 (98.7)	3 (1.3)	0.697 ++
	Spontaneous	162 (97)	5 (3)		164 (98.2)	3 (1.8)	
Nulliparous	No	109 (98.2)	2 (1.8)	0.253 ++	110 (99.1)	1 (0.9)	>0.999 ++
	Yes	276 (95.5)	13 (4.5)		284 (98.3)	5 (1.7)	
Smoking	No	349 (96.1)	14 (3.9)	>0.999 ++	357 (98.3)	6 (1.7)	>0.999 ++
	Yes	36 (97.3)	1 (2.7)		37 (100)	0 (0)	
Pre-existing diabetes	No	374 (96.1)	15 (3.9)	>0.999++	383 (98.5)	6 (1.5)	>0.999 ++
	Yes	11 (100)	0 (0)		11 (100)	0 (0)	
Pre-existing hypertension	No	382 (96.2)	15 (3.8)	>0.999 ++	391 (98.5)	6 (1.5)	>0.999 ++
	Yes	3 (100)	0 (0)		3 (100)	0 (0)	
Systemic lupus erythematosus	No	382 (96.2)	15 (3.8)	>0.999 ++	391 (98.5)	6 (1.5)	>0.999 ++
	Yes	3 (100)	0 (0)		3 (100)	0 (0)	
Antiphospholipid syndrome	No	383 (96.2)	15 (3.8)	>0.999 ++	392 (98.5)	6 (1.5)	>0.999 ++
	Yes	2 (100)	0 (0)		2 (100)	0 (0)	
History of preeclampsia	No	383 (96.2)	15 (3.8)	>0.999 ++	392 (98.5)	6 (1.5)	>0.999 ++
	Yes	2 (100)	0 (0)		2 (100)	0 (0)	
PAPP-A MoM, median (IQR)		1.23 (0.89–1.67)	1.55 (0.82–1.72)	0.852 ‡‡	1.23 (0.89–1.67)	1.48 (0.84–2.07)	0.576 ‡‡
PAPP-A MoM	>5th percentile	365 (96.1)	15 (3.9)	>0.999 ++	374 (98.4)	6 (1.6)	>0.999 ++
	≤5th percentile	20 (100)	0 (0)		20 (100)	0 (0)	
PAPP-A MoM	>10th percentile	348 (96.7)	12 (3.3)	0.181 ++	354 (98.3)	6 (1.7)	>0.999 ++
	≤10th percentile	37 (92.5)	3 (7.5)		40 (100)	0 (0)	
Free β-hCG MoM, median (IQR)		1.01 (0.73–1.5)	1.12 (0.72–1.43)	0.798 ‡‡	1.02 (0.73–1.5)	0.79 (0.67–1.64)	0.655 ‡‡
MAP, mean (SD)		88.8 (8.2)	92.9 (7.7)	0.123 ‡	89 (8.2)	86.7 (10.5)	0.573 ‡
Μean Ut-A PI, mean (SD)		1.48 (0.44)	1.54 (0.5)	0.655 ‡	1.48 (0.44)	1.59 (0.37)	0.600 ‡
Ut-API ≥ 90th perc.	No	324 (96.1)	13 (3.9)	>0.999 ++	333 (98.8)	4 (1.2)	0.408 ++
	Yes	36 (97.3)	1 (2.7)		36 (97.3)	1 (2.7)	
Ut-API ≥95th perc.	No	342 (96.1)	14 (3.9)	>0.999 ++	351 (98.6)	5 (1.4)	>0.999 ++
	Yes	18 (100)	0 (0)		18 (100)	0 (0)	

+ Student’s *t*-test; ++ Fisher’s exact test; ‡ Student’s *t*-test; ‡‡ Mann–Whitney test. Abbreviations: BW: birthweight, BMI: body mass index, underweight: BMI < 18.5, overweight: BMI 25–30, obese: BMI > 30, MAP: mean arterial pressure, Ut-A: uterine artery.

**Table 3:** Association of BW < 10th centile with sample’s maternal characteristics and biochemical and biophysical parameters.

		BW ≤ 10th in One Twin		*p*-Value	BW ≤ 10th Both Twins		*p*-Value
		No (*n* = 341; 85.3%)	Yes (*n* = 59; 14.8%)		No (*n* = 361; 90.3%)	Yes (*n* = 39; 9.8%)	
		*n* (%)	*n* (%)		*n* (%)	*n* (%)	
Age, mean (SD)		35.5 (4.3)	35.9 (4.1)	0.465 ‡	35.6 (4.2)	35.2 (5)	0.537 ‡
Weight, mean (SD)		69.5 (14.8)	68.7 (13.5)	0.698 ‡	69.7 (14.4)	66.8 (16.4)	0.253 ‡
Maternal height, mean (SD)		166 (5.9)	166.2 (6)	0.834 ‡	166.1 (6)	165.3 (5.1)	0.447 ‡
BMI (1st trimester), mean (SD)		25.2 (5)	25 (5.2)	0.740 ‡	25.3 (5)	24.3 (5)	0.270 ‡
Birthweight, mean (SD)		2304.1 (557.7)	2111.7 (385.2)	0.011 ‡	2307.3 (540.9)	1983.2 (435.1)	<0.001 ‡
BMI (1st trimester)	Underweight/Normal	194 (84.3)	36 (15.7)	0.551 +	205 (89.1)	25 (10.9)	0.678 +
	Overweight	98 (88.3)	13 (11.7)		102 (91.9)	9 (8.1)	
	Obese	49 (83.1)	10 (16.9)		54 (91.5)	5 (8.5)	
Chorionicity	Dichorionic	296 (84.8)	53 (15.2)	0.520 +	314 (90)	35 (10)	0.802 ++
	Monochorionic	45 (88.2)	6 (11.8)		47 (92.2)	4 (7.8)	
Conception	IVF	202 (86.7)	31 (13.3)	0.336 +	213 (91.4)	20 (8.6)	0.353 +
	Spontaneous	139 (83.2)	28 (16.8)		148 (88.6)	19 (11.4)	
Nulliparous	No	95 (85.6)	16 (14.4)	0.907 +	102 (91.9)	9 (8.1)	0.493 +
	Yes	246 (85.1)	43 (14.9)		259 (89.6)	30 (10.4)	
Smoking	No	308 (84.8)	55 (15.2)	0.478 +	331 (91.2)	32 (8.8)	0.073 ++
	Yes	33 (89.2)	4 (10.8)		30 (81.1)	7 (18.9)	
Diabetes	No	331 (85.1)	58 (14.9)	>0.999 ++	352 (90.5)	37 (9.5)	0.292 ++
	Yes	10 (90.9)	1 (9.1)		9 (81.8)	2 (18.2)	
Hypertension	No	338 (85.1)	59 (14.9)	>0.999 ++	359 (90.4)	38 (9.6)	0.266 ++
	Yes	3 (100)	0 (0)		2 (66.7)	1 (33.3)	
Systemic lupus erythematosus	No	339 (85.4)	58 (14.6)	0.381 ++	358 (90.2)	39 (9.8)	>0.999 ++
	Yes	2 (66.7)	1 (33.3)		3 (100)	0 (0)	
Antiphospholipid syndrome	No	340 (85.4)	58 (14.6)	0.274 ++	359 (90.2)	39 (9.8)	>0.999 ++
	Yes	1 (50)	1 (50)		2 (100)	0 (0)	
History of preeclampsia	No	339 (85.2)	59 (14.8)	>0.999 ++	359 (90.2)	39 (9.8)	>0.999 ++
	Yes	2 (100)	0 (0)		2 (100)	0 (0)	
PAPP-A MoM, median (IQR)		1.31 (0.92–1.72)	1.09 (0.73–1.36)	0.002 ‡‡	1.26 (0.92–1.68)	1 (0.69–1.59)	0.024 ‡‡
PAPP-A MoM	>5th percentile	323 (85)	57 (15)	0.751 ++	343 (90.3)	37 (9.7)	>0.999 ++
	≤5th percentile	18 (90)	2 (10)		18 (90)	2 (10)	
PAPP-A MoM	>10th percentile	307 (85.3)	53 (14.7)	>0.999 ++	328 (91.1)	32 (8.9)	0.092 ++
	≤10th percentile	34 (85)	6 (15)		33 (82.5)	7 (17.5)	
Free β-hCG MoM, median (IQR)		1.05 (0.74–1.53)	0.9 (0.65–1.21)	0.049 ‡‡	1.03 (0.73–1.5)	0.89 (0.69–1.49)	0.516 ‡‡
MAP, mean (SD)		88.9 (8.2)	89.5 (8.4)	0.665 ‡	88.8 (8.1)	90 (8.8)	0.514 ‡
Μean Ut-A PI, mean (SD)		1.48 (0.44)	1.52 (0.48)	0.559 ‡	1.47 (0.44)	1.59 (0.48)	0.141 ‡
Ut-API ≥90th perc.	No	287 (85.2)	50 (14.8)	0.508 +	307 (91.1)	30 (8.9)	0.370 ++
	Yes	33 (89.2)	4 (10.8)		32 (86.5)	5 (13.5)	
Ut-API ≥95th perc.	No	305 (85.7)	51 (14.3)	0.733 ++	324 (91)	32 (9)	0.231 ++
	Yes	15 (83.3)	3 (16.7)		15 (83.3)	3 (16.7)	

+ Student’s *t*-test; ++ Fisher’s exact test; ‡ Student’s *t*-test; ‡‡ Mann–Whitney test. Abbreviations: BW: birthweight, BMI: body mass index, underweight: BMI < 18.5, overweight: BMI 25–30, obese: BMI > 30, MAP: mean arterial pressure, Ut-A: uterine artery.

**Table 4:** Multiple logistic regression analysis for BW ≤ 10th centile.

Dependent Variables	Independent Variables	OR	95% CI	*p*-Values
BW ≤ 10th in one twin	PAPP-A MoM (per 0.1 MoM)	0.92	0.88–0.98	0.004
BW ≤ 10th both twins	PAPP-A MoM (per 0.1 MoM)	0.94	0.88–0.99	0.041

Abbreviations: BW: birthweight.

**Table 5:** ROC analysis results on the prognostic value of PAPP-A MoM for BW ≤ 3rd centile and BW ≤ 10th centile in one or both twins.

Outcome	AUC	95% CI	*p*-Value	Optimal Cut-Off	Sensitivity (%) 95% CI	Specificity (%) 95% CI	PPV (%) 95% CI	NPV (%) 95% CI
BW ≤ 3rd in one twin	0.49	0.33–0.64	0.852	n/a	n/a	n/a	n/a	n/a
BW ≤ 3rd both twins	0.43	0.18–0.68	0.576	n/a	n/a	n/a	n/a	n/a
BW ≤ 10th in one twin	0.60	0.56–0.70	0.002	<1.408	81.4 (69.1–90.3)	44.6 (39.2–50)	20.3 (15.3–25.9)	93.3 (88.2–96.6)
BW ≤ 10th both twins	0.61	0.51–0.71	0.024	<1.056	61.5 (44.6–79.9)	65.1 (59.6–70)	16.0 (10.5–22.9)	94.0 (90.3–96.6)

Abbreviations: BW: birthweight, n/a: not applicable.

## Data Availability

The original data presented in the study are openly available in Zenodo at DOI 10.5281/zenodo.18002437.

## Supplementary Materials

The following supporting information can be downloaded at https://www.mdpi.com/article/10.3390/life16010149/s1. Figure S1: Centile plots for the full dataset showing six fetal biometric variables across gestational age (15–40 weeks); Table S1: Comparison of the 10th, 50th, and 90th percentiles of estimated fetal weight between the present study and the WHO reference charts at 20, 24, 28, 32, and 36 weeks of gestation. Table S2: Comparison of the distribution of estimated fetal weight measurements across specific centile bands between the study-developed growth charts and WHO reference standards in the test dataset. Table S3: External validation of estimated fetal weight: comparison of the 10th, 50th, and 90th percentiles between the study model and the external dataset across gestation. Table S4: External validation of biparietal diameter: comparison of empirical and model-based centiles across gestation. Table S5: External validation of head circumference: comparison of model-based (Study) and empirical (External) 10th, 50th, and 90th percentiles across gestational ages. Table S6: External validation of femur length: comparison of model-based (Study) and empirical (External) 10th, 50th, and 90th percentiles across gestational ages. Table S7: External validation of abdominal circumference: comparison of model-based (Study) and empirical (External) 10th, 50th, and 90th percentiles across gestational ages. Ref. [38] is cited in Supplementary Materials.

## Abbreviations

The following abbreviations are used in this manuscript:

FGR	Fetal growth restriction
BW	Birthweight
SGA	Small for gestational age
PAPP-A	Pregnancy-associated plasma protein A
b-h CG	Beta-human chorionic gonadotropin
TTTS	Twin-to-twin transfusion syndrome
TAPS	Twin anemia-polycythemia sequence
Ut-A	Uterine artery
MAP	Mean arterial pressure
ART	Assisted reproductive technology
EDD	Estimated due date
DCDA	Dichorionic diamniotic
MCDA	Monochorionic diamniotic
MoM	Multiples of median

## References

[1] Chauhan S.P., Scardo J.A., Hayes E., Abuhamad A.Z., Berghella V. (2010). Twins: Prevalence, problems, and preterm births. Am. J. Obstet. Gynecol..

[2] Sueters M., Oepkes D. (2014). Diagnosis of twin-to-twin transfusion syndrome, selective fetal growth restriction, twin anaemia-polycythaemia sequence, and twin reversed arterial perfusion sequence. Best Pract. Res. Clin. Obstet. Gynaecol..

[3] Committee on Practice Bulletins—Obstetrics, Society for Maternal–Fetal Medicine (2016). Practice Bulletin No. 169: Multifetal Gestations: Twin, Triplet, and Higher-Order Multifetal Pregnancies. Obstet. Gynecol..

[4] Kalafat E., Khalil A. (2022). Assessment of fetal growth in twins: Which method to use?. Best Pract. Res. Clin. Obstet. Gynaecol..

[5] Jahanfar S., Lim K., Ovideo-Joekes E. (2017). Birth weight discordance and adverse perinatal outcomes. J. Perinat. Med..

[6] (2025). America’s Health Rankings analysis of U.S. Department of Health and Human Services; Centers for Disease Control and Prevention; National Center for Health Statistics; National Vital Statistics System; Natality Public Use Files via CDC WONDER Online Database, United Health Foundation. https://AmericasHealthRankings.org.

[7] (2025). National Vital Statistics Reports.

[8] Benkö Z., Wright A., Rehal A., Cimpoca B., Syngelaki A., Delgado J.L., Tsokaki T., De Alvarado M., Vojtassakova D., Ntalianis K.M. (2021). Prediction of pre-eclampsia in twin pregnancy by maternal factors and biomarkers at 11–13 weeks’ gestation: Data from EVENTS trial. Ultrasound Obstet. Gynecol..

[9] Francisco C., Wright D., Benkö Z., Syngelaki A., Nicolaides K.H. (2017). Competing-risks model in screening for pre-eclampsia in twin pregnancy according to maternal factors and biomarkers at 11–13 weeks’ gestation. Ultrasound Obstet. Gynecol..

[10] Queirós A., Domingues S., Gomes L., Pereira I., Brito M., Cohen Á., Alves M., Papoila A.L., Simões T. (2024). First-trimester uterine artery Doppler and hypertensive disorders in twin pregnancies: Use of twin versus singleton references. Int. J. Gynaecol. Obstet..

[11] Queirós A., Gomes L., Pereira I., Charepe N., Plancha M., Rodrigues S., Cohen Á., Alves M., Papoila A.L., Simões T. (2024). First-trimester serum biomarkers in twin pregnancies and adverse obstetric outcomes-a single center cohort study. Arch. Gynecol. Obstet..

[12] Queirós A., Bernardo A., Rijo C., Carocha A., Ferreira L., Martins A.T., Cohen Á., Alves M., Papoila A.L., Simões T. (2025). First-trimester screening and small for gestational age in twin pregnancies: A single center cohort study. Arch. Gynecol. Obstet..

[13] Khalil A., Rodgers M., Baschat A., Bhide A., Gratacos E., Hecher K., Kilby M.D., Lewi L., Nicolaides K.H., Oepkes D. (2016). ISUOG Practice Guidelines: Role of ultrasound in twin pregnancy. Ultrasound Obstet. Gynecol..

[14] Khalil A., Sotiriadis A., Baschat A., Bhide A., Gratacós E., Hecher K., Lewi L., Salomon L.J., Thilaganathan B., Ville Y. (2025). ISUOG Practice Guidelines (updated): Role of ultrasound in twin pregnancy. Ultrasound Obstet. Gynecol..

[15] Lees C., Stampalija T., Baschat A.A., da Silva Costa F., Ferrazzi E., Figueras F., Hecher K., Kingdom J., Poon L.C., Salomon L.J. (2020). ISUOG Practice Guidelines: Diagnosis and management of small-for-gestational-age fetus and fetal growth restriction. Ultrasound Obstet. Gynecol..

[16] Dias T., Arcangeli T., Bhide A., Napolitano R., Mahsud-Dornan S., Thilaganathan B. (2011). First-trimester ultrasound determination of chorionicity in twin pregnancy. Ultrasound Obstet. Gynecol..

[17] Bilagi A., Burke D.L., Riley R.D., Mills I., Kilby M.D., Katie Morris R. (2017). Association of maternal serum PAPP-A levels, nuchal translucency and crown-rump length in first trimester with adverse pregnancy outcomes: Retrospective cohort study. Prenat. Diagn..

[18] Papastefanou I., Wright D., Lolos M., Anampousi K., Mamalis M., Nicolaides K.H. (2020). Competing-risks model for prediction of small-for-gestational-age neonate from maternal characteristics, serum pregnancy-associated plasma protein-A and placental growth factor at 11–13 weeks’ gestation. Ultrasound Obstet. Gynecol..

[19] Morris R.K., Johnstone E., Lees C., Morton V., Smith G., Royal College of Obstetricians and Gynaecologists (2024). Investigation and Care of a Small-for-Gestational-Age Fetus and a Growth Restricted Fetus (Green-top Guideline No. 31). BJOG.

[20] Papastefanou I., Wright D., Nicolaides K.H. (2020). Competing-risks model for prediction of small-for-gestational-age neonate from maternal characteristics and medical history. Ultrasound Obstet. Gynecol..

[21] American College of Obstetricians and Gynecologists (2021). Indications for Outpatient Antenatal Fetal Surveillance: ACOG Committee Opinion, Number 828. Obstet. Gynecol..

[22] Karagiannis G., Akolekar R., Sarquis R., Wright D., Nicolaides K.H. (2011). Prediction of small-for-gestation neonates from biophysical and biochemical markers at 11–13 weeks. Fetal Diagn. Ther..

[23] Sapantzoglou I., Giourga M., Kontopoulou A.M., Pergialiotis V., Daskalaki M.A., Antsaklis P., Theodora M., Thomakos N., Daskalakis G. (2024). Low PAPPA and Its Association with Adverse Pregnancy Outcomes in Twin Pregnancies: A Systematic Review of the Literature and Meta-Analysis. J. Clin. Med..

[24] Swiercz G., Zmelonek-Znamirowska A., Szwabowicz K., Armanska J., Detka K., Mlodawska M., Mlodawski J. (2024). Evaluating the predictive efficacy of first trimester biochemical markers (PAPP-A, fβ-hCG) in forecasting preterm delivery incidences. Sci. Rep..

[25] Saletra-Bielińska A., Kosińska-Kaczyńska K., Szymusik I., Kaczyński B., Brawura-Biskupski-Samaha R., Kozłowski S., Jarmużek P., Walasik I., Wielgoś M. (2020). Both Low and High PAPP-A Concentrations in the First Trimester of Pregnancy Are Associated with Increased Risk of Delivery before 32 Weeks in Twin Gestation. J. Clin. Med..

[26] Kim Y.R., Kim N., Ahn E.H., Jung S.H., Park G., Jung I., Cho H.Y. (2022). The association of maternal serum biomarkers and birth weight in twin pregnancy: A retrospective cohort study. J. Obstet. Gynaecol..

[27] Monod C., Trottmann F., Raio L., Challande P., Amylidi-Mohr S., Musik T., Granado C., Hildebrandt L., Surbek D., de Tejada B.M. (2025). First-trimester combined screening for preeclampsia in twin pregnancies-results of the first 100 twin pregnancies included in the IPSISS (Implementing Preeclampsia Screening in Switzerland) cohort. Am. J. Obstet. Gynecol. MFM.

[28] Rehal A., Benkő Z., De Paco Matallana C., Syngelaki A., Janga D., Cicero S., Akolekar R., Singh M., Chaveeva P., Burgos J. (2021). Early vaginal progesterone versus placebo in twin pregnancies for the prevention of spontaneous preterm birth: A randomized, double-blind trial. Am. J. Obstet. Gynecol..

[29] Trottmann F., Challande P., Manegold-Brauer G., Ardabili S., Hösli I., Schönberger H., Amylidi-Mohr S., Kohl J., Hodel M., Surbek D. (2023). Implementing Preeclampsia Screening in Switzerland (IPSISS): First Results from a Multicentre Registry. Fetal Diagn. Ther..

[30] Rizzo G., Pietrolucci M.E., Aiello E., Capponi A., Arduini D. (2014). Uterine artery Doppler evaluation in twin pregnancies at 11 + 0 to 13 + 6 weeks of gestation. Ultrasound Obstet. Gynecol..

[31] Chen J., Zhao D., Liu Y., Zhou J., Zou G., Zhang Y., Guo M., Duan T., Van Mieghem T., Sun L. (2020). Screening for preeclampsia in low-risk twin pregnancies at early gestation. Acta Obstet. Gynecol. Scand..

[32] Svirsky R., Yagel S., Ben-Ami I., Cuckle H., Klug E., Maymon R. (2014). First trimester markers of preeclampsia in twins: Maternal mean arterial pressure and uterine artery Doppler pulsatility index. Prenat. Diagn..

[33] Kirkegaard I., Uldbjerg N., Oxvig C. (2010). Biology of pregnancy-associated plasma protein-A in relation to prenatal diagnostics: An overview. Acta Obstet. Gynecol. Scand..

[34] Brosens I., Pijnenborg R., Vercruysse L., Romero R. (2011). The “Great Obstetrical Syndromes” are associated with disorders of deep placentation. Am. J. Obstet. Gynecol..

[35] Swiercz G., Zmelonek-Znamirowska A., Szwabowicz K., Armanska J., Detka K., Mlodawska M., Mlodawski J. (2024). Navigating Uncertain Waters: First-Trimester Screening’s Role in Identifying Neonatal Complications. J. Clin. Med..

[36] Wright D., Tan M.Y., O’Gorman N., Syngelaki A., Nicolaides K.H. (2022). Serum PlGF compared with PAPP-A in first trimester screening for preterm pre-eclampsia: Adjusting for the effect of aspirin treatment. BJOG.

[37] Trilla C., Platero J., Mora J., Nan M.N., Medina C., Alejos O., Parra J., Llurba E. (2025). Role of aspirin therapy in modulating uterine artery resistance and placental growth between first and second trimesters of pregnancy. Ultrasound Obstet. Gynecol..

[38] Kiserud T., Piaggio G., Carroli G., Widmer M., Carvalho J., Neerup Jensen L., Giordano D., Cecatti J.G., Abdel Aleem H., Talegawkar S.A. (2017). The World Health Organization Fetal Growth Charts: A Multinational Longitudinal Study of Ultrasound Biometric Measurements and Estimated Fetal Weight. PLoS Med..

