# Supplementary material for: Low PAPP-A Levels and Growth in Twin Pregnancies
Source: Life (Basel). 2026 Jan 16;16(1):149. doi: 10.3390/life16010149 (PMC12843441; doi:10.3390/life16010149)
Supplement: Supplementary file 1 [file life-16-00149-s001.zip › life-4036474-supplementary.pdf]

1 Methods

1.1 Study design and population

This study used data from a prospective cohort from two fetal medicine units (Leto Maternity Hospital and Alexandra Hospital, Athens, Greece). The cohort includes singleton pregnancies receiving routine antenatal care and serial ultrasound examinations between 2012 - 2022. Clinical data were collected from ultrasound scans for assessment of fetal anatomy and growth, while maternal and fetal health indicators were build for analysis. The cohort span of ten years provides a strong basis to construct and evaluate fetal growth reference curves.

1.2 Data sources and variables

The cohort database contains data collected during routine antenatal care. We considered the main fetal biometric variables ie biparietal diameter (BPD), head circumference (HC), transcerebellar diameter (TCD), femur length (FL), abdominal circumference (AC) and estimated fetal weight (EFW). Maternal characteristics were recorded at the initial visit and included age, height, weight, body mass index, gravidity, parity, smoking status, among other clinical information. Such characteristics are reported as descriptive statistics and used for subgroup analysis, while the fetal growth reference charts were based on fetal ultrasound measurements.

1.3 Ultrasound Biometry

BPD, HC, AC, TCD and FL were measured according to local and international protocols [Reference HSOG and ISUOG}]. (1) Estimated fetal weight (EFW) was calculated using the Hadlock IV formula (2):

$$\log(EFW) = 1.3596 + 0.00061(BPD \cdot AC) - 0.00386(AC \cdot FL) + 0.0064\,HC + 0.0424\,AC + 0.174\,FL$$

Each pregnancy contributed with measurements from at least two visits from 16 to 41 gestational weeks. Several women in the cohort participate with more than one pregnancy. The dating of the pregnancy was based on the last menstrual period (LMP) and was corrected at the first trimester if the dates were uncertain or if the difference in fetal growth exceeded one week. All pregnancies had the first trimester ultrasound examination at 11-13 weeks for assessment of risk of chromosomal abnormalities. Fetuses with chromosomal and structural abnormalities and the ones resulting in intra-uterine death were excluded form the analysis. Pregnancy outcome was ascertained from recorded data in the delivery suite or communication with the mother or the referring physician.

1.4 Statistical Modelling

Fetal growth reference charts were constructed via Generalized Additive Models for Location, Scale, and Shape (GAMLSS). GAMLSS allow the distribution of each outcome to vary in its mean, variability, skewness and kurtosis across gestation. This flexibility is vital for fetal biometry, where

visits tend to spread and become skewed in later stages of pregnancy. By modelling these distributional parameters through smoothed functions of gestational age via penalized B-splines, GAMLSS can create a robust framework to construct centile curves.

Six GAMLSS models were fitted, one for each biometric variable. For each, several distributions were evaluated, focusing on families that allow modelling of all four GAMLSS parameters. Model selection was done by the Akaike Information Criterion (AIC). The Johnson's SU (JSU) distribution provided the best or almost best AIC for EFW, HC and TCD, while the Box–Cox *t* (BCT) was a good fit for BPD and FL. The Box–Cox power exponential (BCTo) distribution was chosen for AC. These selections ensured flexibility to capture the growing variability and skewness observed in fetal biometry across gestation. Centile curves (1st, 5th, 10th, 50th, 90th, 95th, 99th) were created showing the expected range of fetal size at each week of gestation.

1.5 Internal Validation

Internal validation was performed using a train–test split of the development dataset. The training subset was used to fit the GAMLSS models, while the test subset was kept for model evaluation. The centile curves of each variable were derived from the training-set models and then applied to the test set observations. Each measurement was assigned to a specific centile band. Calibration was done by comparing the observed distribution of test-set measurements across these centile bands with the proportions expected under the fitted model.

1.6 External Validation

External validation was performed using a separate cohort that included gestational age and the same fetal variables as the initial dataset, except for transcerebellar diameter (TCD) that was only evaluated internally. The external validation assesses the generalizability of curves beyond the population used for model development.

For each of the available biometric parameters (EFW, BPD, HC, FL, AC), the main centile tables were combined with external observations through gestational week. Each measurement was placed in a centile band between the 2.5th and the 97.5th centile and the observed distribution was compared with theoretical proportions expected under the fitted model. This methodological approach is consistent with methods applied in large fetal growth studies, such as WHO and INTERGROWTH-21st, which assessed biometric measurements distributions across centile bands.

1.7 Software

All analyses were performed in R (version 4.3). The GAMLSS package was applied in model fitting and diagnostics. Figures and tables were produced with R and R libraries.

2 Results

2.1 Study Population

The development cohort consisted of 41,812 observations from singleton pregnancies. Their demographic characteristics are presented in Table 1. The dating of the pregnancy was based on LMP and confirmed by the first trimester scan in 93.3% of cases. The mean gestational age at

examination was 27 weeks on average and the six fetal biometric variables showed large differences across gestation, pointing at the anticipated variability in clinical practice.

2.2 Model Fit and Centile Curves

The model fit process identified the JSU distribution as the best fit for EFW, HC, and TCD, the BCT distribution for BPD and FL and the BCTo distribution for AC variable. The centile curves (Figure 1) showed growth patterns similar to expected fetal development, with measurements increasing across gestation and larger variability in later gestational ages. Across all six variables, the curves rose monotonically as gestation advanced and the spread of the upper tail increased across centiles.

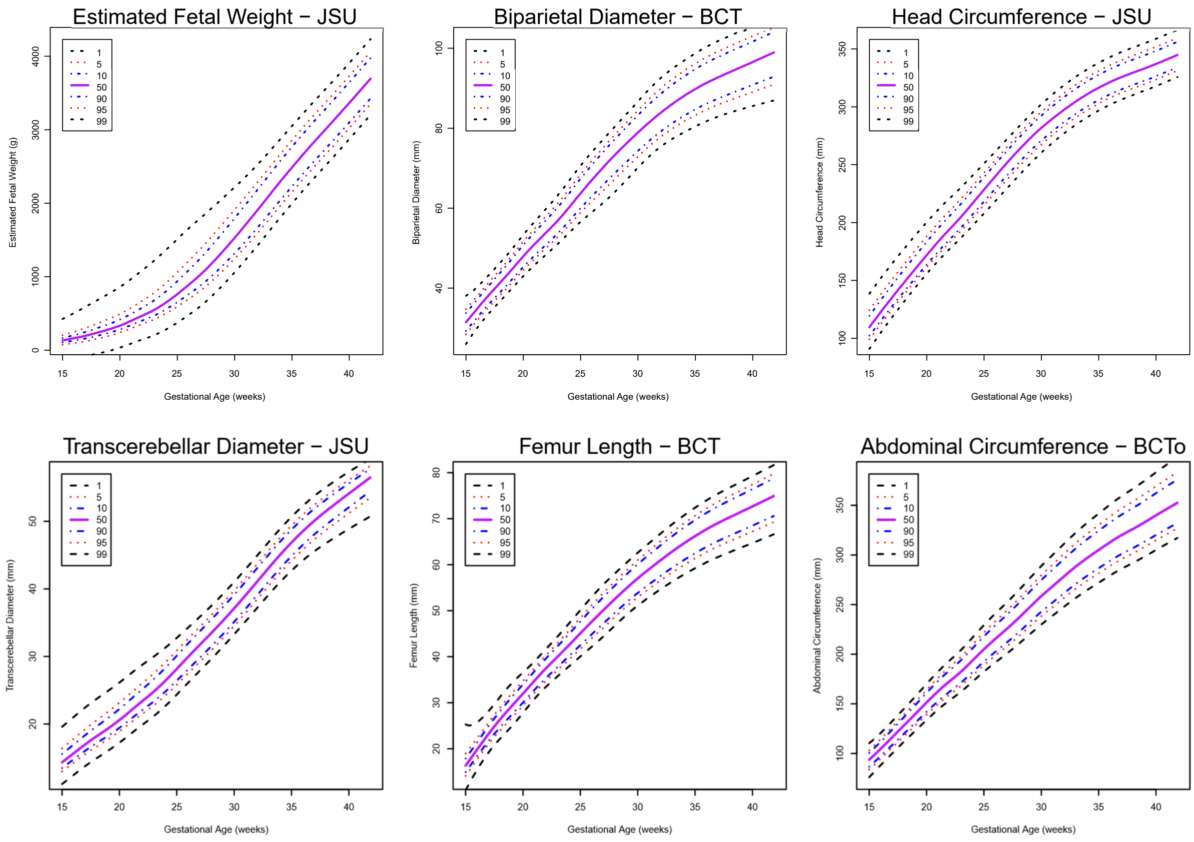

Figure S1: Centile plots for the full dataset showing six fetal biometric variables across gestational age (15–40 weeks): Estimated Fetal Weight (g), Biparietal Diameter (mm), Head Circumference (mm), Transcerebellar Diameter (mm), Femur Length (mm), Abdominal Circumference (mm). Each panel displays the fitted centiles at 1st, 5th, 10th, 50th, 90th, 95th, 99th percentiles, based on the best distribution for each variable (JSU for Estimated Fetal Weight, Head Circumference, and Transcerebellar Diameter, BCT for Biparietal Diameter and Femur Length BCTo for Abdominal Circumference).

2.3 Internal Validation

Internal validation was applied in a temporally independent test subset of the main dataset. All ultrasound examinations conducted before 2022 were used as the training set to fit the GAMLSS models, while examinations from 2022 and after were reserved exclusively for testing .

We compared the centiles to international standards and we checked the agreement between the fitted centiles and the WHO fetal growth charts . For each of the available external variables, the 10th, 50th, 90th percentiles were calculated at 20, 24, 28, 32 and 36 weeks of gestation and compared with the corresponding WHO values. The results for EFW are summarized in Table 2. The differences between this study's and WHO centiles across the examined gestational ages were small with maximum absolute variations staying below 5% (Table 2).

Table S1: Comparison of the 10th, 50th, and 90th percentiles of estimated fetal weight between the present study and the WHO reference charts (Kiserud et al., 2017) at 20, 24, 28, 32, and 36 weeks of gestation.

Reference Chart	20	24	28	32	36
**10th percentile of EFW (g)**					
Study	297.9	569.7	1016.1	1670.1	2319.4
WHO	286.0	576.0	1026.0	1635.0	2352.0
Diff (%)	+4.1%	−1.1%	−1.0%	+2.2%	−1.4%
**50th percentile of EFW (g)**					
Study	336.8	650.3	1177.6	1909.1	2677.0
WHO	330.0	665.0	1189.0	1901.0	2745.0
Diff (%)	+2.1%	−2.2%	−0.9%	+0.4%	−2.5%
**90th percentile of EFW (g)**					
Study	380.9	741.7	1352.1	2166.8	3072.8
WHO	380.0	765.0	1368.0	2187.0	3153.0
Diff (%)	+0.2%	−3.0%	−1.2%	−0.9%	−2.5%

A second assessment examined the distribution of EFW measurements across predefined centile bands using both the present study's and the WHO reference curves. We present the results for EFW in Table 3. As shown in Table 3, the proportions of EFW measurements that fall in each centile band were very similar between the two reference curves. Only minor differences can be found and mostly in extreme bands.

Table S2: Comparison of the distribution of estimated fetal weight (EFW) measurements across specific centile bands between the study-developed growth charts and WHO reference standards (Kiserud et al. 2017) in the test dataset.

Band	N_{Study}	N_{WHO}	% Study	% WHO	Diff (%)
<2.5	31	14	2.8	1.3	1.5
2.5–5	22	17	2.0	1.5	0.5
5–10	67	66	6.0	6.0	0.0
10–25	147	163	13.3	14.7	-1.4
25–50	285	302	25.7	27.3	-1.6
50–75	264	262	23.8	23.6	0.2
75–90	152	175	13.7	15.8	-2.1
90–95	75	55	6.8	5.0	1.8
95–97.5	28	31	2.5	2.8	-0.3
>97.5	38	23	3.4	2.1	1.3
**Total**	**1109**	**1108**			

2.4 External Validation

In the external validations the study charts showed good transportability across all five biometric variables. The external percentiles of EFW were generally close to the model predictions but the size of the differences decreased with advancing gestational age (Table 4).

The external percentiles of the other parameters are presented in Tables 5-7.

Table S3: External validation of estimated fetal weight (EFW): comparison of the 10th, 50th, and 90th percentiles between the study model and the external dataset across gestation.

GA (weeks)	Study p10	Study p50	Study p90	Ext p10	Ext p50	Ext p90	N
20	297.8	336.6	380.8	331.0	381.0	429.4	103
24	569.4	650.0	741.4	613.4	692.5	789.4	80
28	1016.2	1177.8	1351.8	1087.4	1246.0	1395.8	215
32	1670.0	1909.4	2166.9	1761.8	2001.0	2295.2	955
36	2318.9	2676.8	3073.7	2354.1	2679.0	3088.5	122

Table S4: External validation of biparietal diameter (BPD): comparison of empirical and model-based centiles across gestation.

GA (weeks)	Study p10	Study p50	Study p90	Ext p10	Ext p50	Ext p90	N
20	45.16	47.99	50.92	47.14	49.70	52.60	103
24	56.65	60.24	63.90	56.98	61.40	64.70	80
28	68.59	72.93	77.36	68.30	73.10	78.20	215
32	79.86	84.79	89.86	80.09	85.70	90.60	955
36	87.48	92.67	97.96	86.81	92.90	98.40	122

Table S5: External validation of head circumference (HC): comparison of model-based (Study) and empirical (External) 10th, 50th, and 90th percentiles across gestational ages.

GA (weeks)	Study p10	Study p50	Study p90	Ext p10	Ext p50	Ext p90	N
20	164.06	171.96	180.45	170.02	179.50	189.42	103
24	206.97	216.70	227.07	211.56	220.85	231.23	80
28	249.93	262.47	273.68	257.04	268.10	277.90	215
32	285.64	297.52	310.15	289.38	301.60	314.42	955
36	309.06	321.48	334.83	311.59	324.70	338.20	122

Table S6: External validation of femur length (FL): comparison of model-based (Study) and empirical (External) 10th, 50th, and 90th percentiles across gestational ages.

GA (weeks)	Study p10	Study p50	Study p90	Ext p10	Ext p50	Ext p90	N
20	29.88	32.01	34.19	31.70	33.70	36.10	103
24	40.06	42.52	44.97	41.09	43.10	45.91	80
28	49.65	52.53	55.36	50.30	52.90	55.30	215
32	58.04	61.02	63.90	58.80	61.30	64.10	955
36	64.37	67.73	70.92	63.94	67.05	70.30	122

Table S7: External validation of abdominal circumference (AC): comparison of model-based (Study) and empirical (External) 10th, 50th, and 90th percentiles across gestational ages.

GA (weeks)	Study p10	Study p50	Study p90	Ext p10	Ext p50	Ext p90	n
20	141.62	151.40	161.39	149.62	160.40	168.34	103
24	181.48	193.53	206.20	187.80	199.20	211.62	80
28	221.80	236.16	251.20	229.72	242.50	254.94	215
32	262.99	278.60	294.91	268.64	282.60	299.60	955
36	293.85	312.85	333.26	294.70	311.95	333.95	122
